# Narrative Review of Anomalous Origin of Coronary Arteries: Pathophysiology, **Management, and Treatment**

**DOI:** 10.2174/1573403X19666230530095341

**Published:** 2023-10-02

**Authors:** Sai Gautham Kanagala, Vasu Gupta, Garrett V Dunn, Harmanjit Kaur, Farid Zieneddine, Rohit Jain, Nikita Garg

**Affiliations:** 1 Osmania Medical College, Hyderabad, Telangana 500095, India;; 2 Dayanand Medical College and Hospital, Ludhiana, India;; 3 Pennsylvania State College of Medicine, Hershey, Pennsylvania-17033, United States;; 4 Government Medical College, Patiala, India;; 5 Department of Internal Medicine, Penn State Milton S Hershey Medical Center, Hershey, PA-17033, United States;; 6 Penn State Health Milton S. Hershey Medical Center, Hershey, PA, USA;; 7 Department of Pediatrics, SIU School of Medicine, Springfield, Illinois, USA

**Keywords:** Coronary artery anomalies, sudden cardiac death, left aortic sinus, right coronary sinus, the anomalous aortic origin of the coronary artery, anomalous coronary arteries from the pulmonary artery

## Abstract

Coronary artery anomalies (CAA) are a diverse group of congenital anomalies and are the second most common cause of sudden cardiac death in the young population after Hypertrophic Cardiomyopathy (HCM). Symptoms range from chest pain, syncope, or sudden cardiac arrest to completely asymptomatic. The prevalence of congenital coronary artery anomalies in the general population is estimated to be between 1% and 2%. CAA often gets underdiagnosed due to the lack of knowledge of the disease process. Approximately 5% of patients with acute myocardial infarction do not have atherosclerotic coronary artery disease or luminal narrowing due to other causes. Congenital coronary artery anomalies account for 50-60% of this 5% of patients. Most patients are asymptomatic for most of their lives, and chest pain is the most common symptom in symptomatic patients when referred for coronary angiography, typically when the diagnosis is typically made. The malignant coronary artery is a rare presentation of a coronary anomaly when associated with atherosclerotic coronary artery disease or valvular heart disease. Patients with symptoms of an abnormal coronary artery origin will receive medical treatment/observation, exercise restriction, coronary angioplasty with stent deployment, or surgical repair.

## 1 INTRODUCTION

The coronary arteries normally arise from aortic sinuses, also known as the sinuses of Valsalva, with the right coronary artery (RCA) emerging from the right coronary sinus and the left coronary artery (LCA) arising from the left coronary sinus [1]. Congenital coronary artery anomalies result from abnormal coronary embryogenesis [2]. Due to the rarity of coronary artery anomalies and their association with sudden cardiac death, it is essential to identify them as early as possible [3]. To better understand coronary artery anomalies (CAAs), it is necessary first to comprehend the various types of CAAs and their risk of association with sudden cardiac death [4]. There have been numerous classification systems of anomalous coronary arteries. The most common categorical method is that of the ectopic origin site, which involves the ectopic origin of a coronary artery from one of the following locations as shown in Figs. (**1** and **2**): 1) Anomalous coronary artery from the pulmonary artery (ACAPA); 2) Anomalous aortic origin of coronary artery (AAOCA); 3) Congenital atresia of the left main artery [5]. The most common type is the ectopic aortic origin, with an anomalous origin of the coronary artery from the opposite sinus of Valsalva [6]. The course of CAA is crucial to identify when dealing with anomalous origins from the opposite sinus. CAAs are classified into five subtypes based on their course: interarterial, subpulmonic, prepulmonic, retroaortic, or retrocardiac [5]. In the interarterial course of the disease, the prognosis is more severe, and the risk of SCD is increased due to the risk of compression of the anomalous artery between the great vessels. One theory is that exercise causes aortic root and pulmonary trunk expansion, which may increase the existing angulation of the coronary artery, reducing the luminal diameter [4]. Abnormal aortic origin of coronary arteries(AAOCA) with an interarterial course has been reported to occur in families. Screening first-degree relatives in patients with a coronary anomaly may be a realistic practice, mainly if the condition presents as sudden death [7]. When presenting with an interarterial course, adults are believed to have a better prognosis than young patients [8]. In the case of anomalous coronary arteries from the pulmonary artery (ACAPA), all coronary arteries originate from the pulmonary artery. In this anomaly, the pulmonary artery supplies the entire coronary circulation and has a poor prognosis, and most patients die within the first month of life. ARCAPA (abnormal origin of the right coronary artery from the pulmonary artery) is a rare congenital anomaly of the coronary arteries where the right coronary artery originates from the pulmonary trunk. It is estimated to have a prevalence of 0.002%, which is slightly less common than ALCAPA (anomalous left coronary artery from the pulmonary artery) with an incidence of 0.008%. ARCAPA typically presents later in life and is less likely to cause congestive heart failure, myocardial ischemia, or sudden cardiac death than its left-sided counterpart [9].

ARCAPA is most commonly associated with the aortopulmonary window (30%) and tetralogy of Fallot (19%) [10]. Most patients with these anomalous coronary variants are asymptomatic. However, symptomatic patients may present with anginal chest pain or syncope, especially with strenuous exercise. Sometimes, patients may present with sudden cardiac death as the initial symptom, particularly in the younger population. Myocardial functional studies are frequently used for risk stratification during suggestive testing to look for evidence of inducible ischemia. Some patients may require invasive cardiac catheterization, including intravascular ultrasound (IVUS) and fractional flow reserve (FFR) measurement. Over the last decade, efforts have been made to advance knowledge about the anomalous origin of coronary arteries (AOCA), including anatomic types, diagnostic evaluation, and management strategies. This article aims to provide current information about epidemiology and various methods and strategies used to diagnose the condition and manage patients [11].

## 2 PATHOPHYSIOLOGY

ALCAPA is caused by either aberrant conotruncus septation into the aorta and pulmonary artery or by the persistence of the pulmonary buds in conjunction with the involution of the aortic buds, which together create the coronary arteries [12]. It is divided into two types: infant and adult. The infant type manifests early due to little or limited collateral artery development between the RCA and the LCA, resulting in reduced blood supply to the left heart, heart failure, and sudden cardiac death. In the adult type, symptoms manifest secondary to the steal phenomenon in which blood shunts from the high-pressure LCA to the low-pressure PA, which induces chronic ischemia that manifests as dilated cardiomyopathy (DCM), mitral regurgitation, chest pain, dyspnea on exertion, or acute coronary syndrome and ventricular arrhythmia [13]. Infantile patients die weeks to months after birth without surgical correction, while 80-90% of adults experience SCD by a mean age of 35 [14]. It often presents alone; however, concomitant anomalies such as coarctation of the aorta, atrial septal defect, and ventricular septal defect have been documented [12]. AAOCA is an abnormality of origin and course of the coronary artery and anomalous origin of RCA or LCA from the opposite sinus of Valsalva. It is the most common and clinically relevant entity [15]. The development of the coronary arteries is a complex process with many locations where mutations can potentially lead to an anomalous origin [16]. Animal studies have shown that multiple, rather than a single gene mutation, might be responsible for AAOCA, although the exact mechanism remains unclear [16]. The various anatomical subtypes of AOCA are summarized in Fig. (**3**) below.

## 3 CLINICAL FEATURES

AOCA can cause a wide range of symptoms, from angina, syncope, heart failure, and sudden cardiac death to no symptoms [17]. Sudden cardiac death (SCD) is an unexpected death caused by a heart condition that occurs within one hour of symptoms and accounts for almost 15-20% of deaths in Western countries [18]. AOCA is a rare cause of SCD, accounting for 19% of sudden deaths in young athletes [19]. Although there is no clear mechanism, it is hypothesized that increased blood flow through the aorta compresses the anomalous coronary artery during strenuous exercise, causing ischemia [20]. In addition, chronic subclinical myocardial hypoperfusion leads to fibrosis, which is potential foci of ventricular arrhythmia.

## 4 DIAGNOSIS

Sudden cardiac death and cardiac ischemia are both found to be related to AOCA, so the establishment of reliable diagnostic methods is a major focus to improve its underdiagnosis. Coronary computed tomography angiography (CCTA) and cardiac magnetic resonance imaging (CMRI) are currently the primary modalities for imaging in patients with diagnosed or clinically suspected anomalous coronary artery origin. Both have a high spatial resolution. CCTA is most commonly used by institutions and has better visualization than CMRI of some high-risk features, including ostia with slit-like origins and acute take-off angle, as well as both systolic and diastolic proximal narrowing [21]. CMRI has slightly lower spatial resolution than CCTA and does not visualize the origins of anomalous arteries accurately or precisely. However, it offers benefits in evaluating valvular and ventricular function, regional contractility, and myocardial viability [22]. It also does not require the use of contrast or exposure of the patient to ionizing radiation, making it particularly useful in pediatric populations. Echocardiography is one option for identifying AOCA as it is inexpensive, readily accessible, does not expose the patient to radiation, and has been used to identify the Ostia and the intramural and extramural course of the arteries. However, its utility is restricted in adults or patients with limited transthoracic acoustic windows, and its quality relies on the sonographer's experience [22]. Lorber *et al.* studied 159 patients with AOCA, comparing institutional reports to external ultrasonographer findings. It was reported that there was a poor correlation, indicating the need for echocardiography protocols when diagnosing AOCA [23]. Other available imaging modalities include single-photon emission computed tomography (SPECT) and positron emission tomography (PET), both primarily useful for evaluating ischemia in patients. Invasive coronary angiography is another option but is no longer utilized as a first-line option due to its invasive nature and inability to visualize much of cardiac anatomy. However, IVUS does provide a clear evaluation of coronary artery anatomy, stenosis severity, and length of the intramural course, so it may be used for risk stratification, especially in cases where imaging lacks sufficient details [3].

## 5 TREATMENT

It is imperative to diagnose and treat CAA as it is the second most common cause of SCD in young athletes in the United States of America [19]. The management strategies for the AOCA are controversial and vary diversely across different institutions throughout the country [24], and include a conservative approach with or without exercise restriction, medical management using beta-blockers, and surgical intervention [21]. Firstly, diagnostic imaging and exercise stress tests should be performed before participating in competitive sports. The European Society of Cardiology recommends that participation may be considered in asymptomatic patients with a negative stress test and no high-risk anatomical features such as intra-mural course, slit-like orifice, or acute take-off angle orifice>1cm. Participation should be avoided for at least three months after a surgical repair of AOCA. It may be resumed if there is no evidence of induced ischemia or cardiac arrhythmias during a stress test [25]. Furthermore, medical management using beta-blockers or calcium channel blockers may help by reducing the left ventricular contractility and thus decreasing the mechanical compression on the anomalous arteries. However, it does not improve patient symptomatology, and no substantial data is available to support this evidence [26]. Lastly, surgical management has been a topic of debate for a long time, and according to the 2020 guidelines released by the European Society of Cardiology, indication for surgery depends on the anatomical subtypes, presence or absence of high-risk anatomical features, and inducible myocardial ischemia Table **1** [27]. According to 2018 AHA/ACC guidelines for the management of adults with congenital heart disease, surgical intervention is indicated in both left and right AAOCA (Class 1 level of recommendation) if the patient presents with symptoms of ischemia and if ischemia is induced during diagnostic testing. In the case of asymptomatic left AAOCA and right AAOCA (with ventricular arrhythmias), surgical intervention is indicated as a Class 2a level of recommendation. Observation is indicated in the right AAOCA without any symptoms or ventricular arrhythmias [28].

Fractional flow reserve (FFR) and instantaneous wave-free ratio (IFR) are two techniques used to manage the anomalous origin of coronary arteries. FFR measures the pressure in the coronary artery before and after the blockage. In contrast, IFR measures the pressure during a specific part of the cardiac cycle when blood flow is most constant. These techniques help identify whether the blockage is causing a significant reduction in blood flow to the heart muscle and guide the decision-making process for further interventions such as medical therapy, percutaneous coronary intervention, or surgical revascularization. FFR and IFR can aid in the individualized treatment of patients with anomalous origin of coronary arteries, improving their outcomes and quality of life. Besides these, other strong determinants for surgery are patient age, a history of an aborted sudden cardiac death, exercise-induced syncope, chest pain, and amount of physical activity [29-31]. The clinicians usually prefer surgical reimplantation of the anomalous coronary artery to the aorta in all cases of ACAPA to reverse the left to right shunt and prevent any long-term complications like cardiac remodeling, arrhythmias, coronary steal phenomenon, and SCD [32]. Various surgical procedures are available for AAOCA, including unroofing, coronary translocation, creation of a neo-ostium, and pulmonary translocation [11]. The overall surgical success rate is high, with no known occurrences of SCD, although postoperative complications are reported to be 7-20%, with up to 15% of patients remaining symptomatic [33].

## CONCLUSION

Coronary artery anomalies represent a heterogeneous and varied set of congenital disorders exhibiting a wide range of clinical manifestations and pathophysiological mechanisms. Due to the complexity of these anomalies, it is crucial to approach their management on an individualized basis. The anatomy of anomalous coronary arteries can vary greatly between patients, and each case requires careful evaluation to determine the optimal treatment plan. Factors such as the location of the anomalous vessel, the extent of any associated cardiac abnormalities, and the patient's overall health and medical history must all be taken into consideration when developing a management strategy. By tailoring the approach to each patient's unique circumstances, healthcare professionals can optimize outcomes and reduce the risk of complications associated with these challenging cardiac defects. Cardiologists also need to develop a broader database in the diagnosis and management of coronary artery anomalies, particularly in the context of advising individuals with CAA who engage in sports or military activities. This will enable them to provide competent and effective guidance to their patients. As our understanding of these conditions continues to grow, we can expect to see improved outcomes and better quality of life for patients affected by these conditions.

## Figures and Tables

**Fig. (1):**
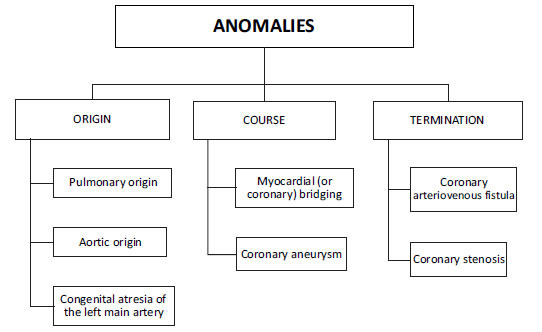
Types of coronary artery anomalies [6].

**Fig. (2):**
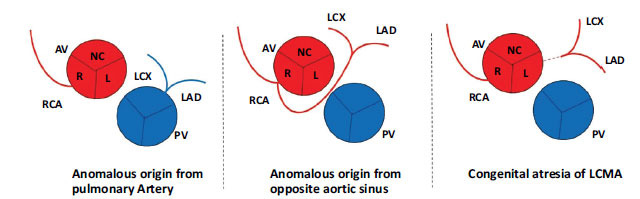
Types of coronary artery anomalies based on ectopic origin.

**Fig. (3):**
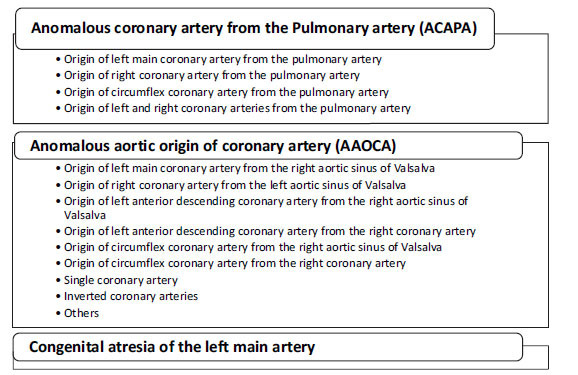
Classification of Anomalous Coronary arteries based on origin [5].

**Table 1:** Summary of management of AOCA [29].

**2020 Guidelines of European Society of Cardiology**
**ACAPA (Anomalous coronary artery from pulmonary artery)** • Surgery is recommended in patients with ALCAPA and symptomatic ARCAPA. • Surgery should be considered in asymptomatic ARCAPA with ventricular dysfunction or myocardial ischemia due to coronary anomaly.
**AAOCA (Anomalous aortic origin of coronary artery)** • Surgery is recommended in patients if AOCA in patients with angina symptoms and evidence of stress-induced myocardial ischemia or high-risk anatomical features.* • Surgery should be considered in asymptomatic AAOLCA or AAORCA with evidence of myocardial ischemia. • Surgery should be considered in asymptomatic AAOLCA with no evidence of myocardial ischemia but high-risk anatomy. • Surgery may be considered in symptomatic AAOCA without any evidence of myocardial ischemia or high-risk anatomy. • Surgery may be considered in asymptomatic AAOLCA without any evidence of myocardial ischemia or high-risk anatomy but presenting at a young age (<35 years) • Surgery is not recommended in asymptomatic AAORCA without any evidence of myocardial ischemia or high-risk anatomy.

## LIST OF ABBREVIATIONS

CAA: Coronary Artery Anomalies
RCA: Right Coronary Artery
LCA: Left Coronary Artery
ACAPA: An Anomalous Coronary Artery from Pulmonary Artery
AAOCA: Anomalous Aortic Origin of Coronary Artery
ALCAPA: Anomalous Origin of the Left Coronary Artery from the Pulmonary Artery
ARCAPA: Anomalous Origin of the Right Coronary Artery from the Pulmonary Artery
AOCA: Anomalous Origin of Coronary Arteries
DCM: Dilated Cardiomyopathy
HCM: Hypertrophic Cardiomyopathy
SCD: Sudden Cardiac Death
IVUS: Intravascular Ultrasound
CCTA: Coronary Computed Tomography Angiography
CMRI: Cardiac Magnetic Resonance Imaging
SPECT: Single-Photon Emission Computed Tomography
PET: Positron Emission Tomography
FFR: Fractional Flow Reserve
IFR: Instantaneous Wave-Free Ratio
